# TGFBI remodels adipose metabolism by regulating the Notch-1 signaling pathway

**DOI:** 10.1038/s12276-023-00947-9

**Published:** 2023-03-01

**Authors:** Seul Gi Lee, Jongbeom Chae, Seon Min Woo, Seung Un Seo, Ha-Jeong Kim, Sang-Yeob Kim, David D. Schlaepfer, In-San Kim, Hee-Sae Park, Taeg Kyu Kwon, Ju-Ock Nam

**Affiliations:** 1 Department of Food Science and Biotechnology, Kyungpook National University, Daegu, 41566 Republic of Korea; 2 Department of Immunology, School of Medicine, Keimyung University, Daegu, 42601 Republic of Korea; 3 Department of Physiology, School of Medicine, Kyungpook National University, Daegu, 41944 Republic of Korea; 4 ASAN Institute for Life Sciences, ASAN Medical Center, Seoul, 05505 Republic of Korea; 5 Moores Cancer Center, University of California, San Diego, La Jolla, CA 92093 USA; 6 KU-KIST Graduate School of Converging Science and Technology, Korea University, Seoul, 02841 Republic of Korea; 7 Center for Theragnosis, Biomedical Research Institute, Korea Institute Science and Technology (KIST), Seoul, 02792 Republic of Korea; 8 School of Biological Sciences and Technology, Chonnam National University, Gwangju, 61186 Republic of Korea; 9 Center for Forensic Pharmaceutical Science, Keimyung University, Daegu, 42601 Republic of Korea; 10 Research Institute of Tailored Food Technology, Kyungpook National University, Daegu, 41566 Republic of Korea

**Keywords:** Obesity, Mechanisms of disease

## Abstract

Extracellular matrix proteins are associated with metabolically healthy adipose tissue and regulate inflammation, fibrosis, angiogenesis, and subsequent metabolic deterioration. In this study, we demonstrated that transforming growth factor-beta (TGFBI), an extracellular matrix (ECM) component, plays an important role in adipose metabolism and browning during high-fat diet-induced obesity. TGFBI KO mice were resistant to adipose tissue hypertrophy, liver steatosis, and insulin resistance. Furthermore, adipose tissue from TGFBI KO mice contained a large population of CD11b^+^ and CD206^+^ M2 macrophages, which possibly control adipokine secretion through paracrine mechanisms. Mechanistically, we showed that inhibiting TGFBI-stimulated release of adipsin by Notch-1-dependent signaling resulted in adipocyte browning. TGFBI was physiologically bound to Notch-1 and stimulated its activation in adipocytes. Our findings revealed a novel protective effect of TGFBI deficiency in obesity that is realized via the activation of the Notch-1 signaling pathway.

## Fat metabolism: protecting against obesity-related metabolic disorders

Studying a protein called TGFBI, which regulates adipose expansion, may help the development of new approaches to protect against obesity and related metabolic disorders such as type II diabetes. White adipose tissue (body fat) stores energy, and brown adipose tissue generates heat. Promoting “browning” of adipose tissue may help protect against obesity. Proteins that influence adipose microenvironment can be involved in metabolic diseases, so Seul Gi Lee and Ju-Ock Nam at Kyungpook National University, Taeg Kyu Kwon at Keimyung University, both in Daegu, South Korea, and co-workers investigated how TGFBI affects adipose metabolism in a mouse model. Deleting TGFBI in mice promoted the transformation of white adipose tissue to brown, protecting mice against weight gain and increase in adipose tissue. These results offer insights into potential therapies for obesity and related disorders via TGFBI regulation.

## Introduction

Obesity is a major health concern worldwide that is associated with several diseases, including cerebrovascular disease, hypertension, hyperlipidemia, and type II diabetes^1^. In most countries, the incidence of obesity and obesity-induced metabolic diseases has been increasing^2,3^. White adipose tissue (WAT) plays an important role in maintaining energy balance, including glucose homeostasis and lipid metabolism, by storing calories and secreting adipokines, such as leptin and resistin^4,5^. Several WATs contain thermogenic adipocytes (brite or beige adipocytes) that transform energy-storing white adipocytes into heat-producing beige adipocytes upon acute cold exposure^6,7^. Thus, the browning of WAT is considered a potential strategy for treating obesity.

Adipocytes are in constant contact with a network of insoluble proteins and polysaccharides that are part of the extracellular matrix (ECM)^8^. Changes in ECM deposition indicate hyperplastic development in adipose tissue and are associated with reduced tissue plasticity^9^. The ECM microenvironment stimulates adipocyte differentiation in adult human bone marrow mesenchymal stem cells (BM-MSCs)^10^.

Transforming growth factor-beta-induced protein (TGFBI, also known as βig-H3 or keratoepithelin) is an ECM protein whose expression is upregulated by TGF-β1, a regulator of angiogenesis and new blood vessel formation in various tumors. We recently reported that TGFBI deletion promotes adipose angiogenesis by regulating essential processes in endothelial cells^11^. Angiogenic factors affect metabolic diseases, such as obesity and diabetes^12,13^. Based on the contribution of TGFBI to adipose angiogenesis, we investigated whether TGFBI functions as a regulator of chronic processes, such as adipose expansion and inflammation, and whether it can control metabolic health during obesity.

Notch-1 activation is an important process in tumor angiogenesis that regulates the responsiveness of endothelial cells to vascular endothelial growth factor (VEGF)^14^. Several studies have indicated that Notch-1 signaling is sufficient to induce the differentiation and tumorigenic transformation of mature adipocytes^15,16^; however, the precise mechanisms underlying the ability of Notch-1 to regulate gene expression remain unclear.

In the present study, we determined the role of TGFBI in adipose tissue expansion and related metabolic disorders using a mouse model of high-fat diet (HFD)-induced obesity. We found that TGFBI KO mice were protected against obesity, as shown by the limited adipose expansion and improved insulin/glucose homeostasis. Mechanistically, TGFBI directly bound to Notch-1 and subsequently regulated adipsin secretion. Our findings establish the importance of TGFBI in regulating adipose metabolism in obesity and present the mechanisms underlying this effect.

## Materials and methods

### Generation of TGFBI KO mice

Generation and genotyping of TGFBI ^−/−^ (KO) mice with the C57/BL6 background was performed as previously described^11^. The TGFBI-targeting vector was constructed with sites introduced upstream and downstream of exon 3, as shown in Supplementary Fig. 1a. The target plasmid was microinjected into C57BL/BL6 blastocysts, and the FLP recombinase target-flanked PGK-neomycin cassette was removed by Flp-mediated recombination. Genotypes were determined by tail biopsy using polymerase chain reaction (PCR). The following primers were used: sense (5’-CCATACTCTGACTTCCAGGTTATTA-3’) and antisense (5’-TGGCAGACTAGCAAGGGTTT-3’). All animals were maintained in a controlled environment with 10–20% humidity, a 24 ± 1 °C temperature, and a 12-h light/dark cycle. The mice were provided free access to water and fed either a standard chow diet or a 60% HFD. All animal experiments were approved by the Institutional Animal Care Committee of Kyungpook National University (approval number: KNU 2017–0059). The study was performed in compliance with the ARRIVE guidelines, and all methods were implemented according to relevant guidelines and regulations.

### GTT and ITT

After 10 h of fasting, glucose and insulin tolerance tests (GTTs and ITTs, respectively) were performed on 20-week-old mice fed an HFD for 12 weeks. For the GTT and ITT, the mice were injected intraperitoneally with either D-glucose (1 g/kg) or insulin (1 unit/kg). Glucose levels were measured from tail bleeds at 0, 15, 30, 60, 90, and 120 min using an AccuChek-EZ glucose monitor (Roche Molecular Biochemicals, IN, Indiana).

### Recombinant TGFBI

Recombinant TGFBI expression was induced and extracted from engineered *Escherichia coli* as previously described^17,18^. The TGFBI cDNA (complementary DNA) was inserted into the *Eco*RV and *Eco*RI sites of pET-29b (Novagen, Madison, WI, USA). A clone was selected, cultured, and induced with isopropyl ß-D-1-thiogalactopyranoside (IPTG) to express recombinant TGFBI protein. The recombinant protein was purified using a Ni-NTA column, dialyzed, subjected to sodium dodecyl sulfate‒polyacrylamide gel electrophoresis (SDS‒PAGE), and stained with Coomassie brilliant blue staining solution.

### Culture and differentiation of 3T3-L1 adipocytes

Mouse 3T3-L1 preadipocytes were purchased from the Korea Cell Line Bank (Seoul, Korea). The cells were maintained in Dulbecco’s modified Eagle’s medium (DMEM) with 10% NBCS and antibiotics (1% penicillin/streptomycin) at 37 °C in a humidified incubator supplied with 5% CO_2_. After reaching 100% confluence, the cells were maintained for another 2 days and then exposed to differentiation medium (MDI) consisting of DMEM with 10% FBS, 0.5 mM IBMX, 10 µg/ml insulin, 1 µM dexamethasone, and 100 µM indomethacin. After 2 days, the culture medium was replaced with DMEM with 10% FBS and insulin and changed every 2 days thereafter (from Day 2 to Day 8).

### Transfection

Lentivirus-expressing small interfering RNAs (siRNAs) against TGFBI (Cat. no. 4659409) and lentiviral scrambled siRNA (Cat. no. LV015-G) were purchased from ABM. Then, 293FT cells were transfected with a lentiviral vector plasmid together with each of the packaging plasmids. After transfection, the viruses were harvested and passed through a 0.4**-**μm filter for infection. Then, 3T3-L1 cells were infected with the virus in the presence of polybrene in 6-well plates. After two days, the medium containing the virus was removed and replaced with fresh medium. Subsequently, the cells were selected continuously using puromycin (1–2 µg/ml).

### Isolation of BM-MSCs and BM-DMs

BM-MSCs and bone marrow-derived macrophages (BM-DMs) were isolated from the hind legs of 6–8-week-old WT and TGFBI KO mice as previously described^19,20^, with some modifications (BM isolation and BM-DM isolation). For BM-MSC differentiation into beige adipocytes, the isolated cells were treated with an adipogenic cocktail (0.5 mM IBMX, 170 nM insulin, 5 µM dexamethasone, 125 µM indomethacin, 2 nM T3, and 1 µM rosiglitazone). After two days, the differentiation medium was replaced with DMEM with 170 nM insulin, 2 nM T3, and 1 µM rosiglitazone for 10 days. Isolated BM-DMs were cultured in DMEM containing 30% L929 conditioned medium and 20% FBS. A week after isolation, the BM-DM culture medium was collected and centrifuged at 500 × *g* for 10 min at 4 °C to remove cell debris. The resulting macrophage-conditioned medium (M-CM) was used at a 1:1 ratio with growth medium containing an adipogenic cocktail. Mature adipocytes from BM-MSCs cultured without M-CM were used as controls.

### Flow cytometry

Infiltrated macrophages were isolated from inguinal white adipose tissue (iWAT) of WT and TGFBI KO mice. iWAT tissues were dissociated with collagenase type I and centrifuged to isolate the stromal vascular fractions (SVFs). SVFs were used for the purification of CD45 microbead-labeled cells. Magnetic-activated cell sorting was performed according to the manufacturer’s instructions (Miltenyi Biotec, Bergisch Gladbach, Germany). Subsequently, CD45 + microbead-labeled cells were stained with the following antibodies: Cy7-CD45 (1:200), FITC-CD11b (1:200), and APC-CD206 (1:200). Cells were washed with phosphate-buffered saline (PBS) and then analyzed using a FACSCanto II (BD Biosciences, Franklin Lakes, NJ, USA) cytometer.

### Cytokine array

Cell culture supernatants of BM-DMs isolated from WT and KO mice were collected, and a cytokine array was performed using a mouse cytokine array kit in accordance with the manufacturer’s instructions (Cat. ab133993, Abcam, Cambridge, UK). Twenty-two cytokines were screened; detailed gene coordinates are available online (http://www.abcam.com/cytokines-array--mouse-cytokines-antibody-array).

### In vitro Notch binding assay

Recombinant Notch-1 protein (2 g/ml) in PBS was coated onto 96-well polystyrene microplates at room temperature (RT) overnight. The plates were washed and blocked with 1% bovine serum albumin (BSA) in PBS for 1 h at RT. The blocking solution was removed, and the binding proteins Jagged-1 or TGFBI were added at concentrations ranging from 0 to 2000 ng/ml. After incubation, biotinylated antibodies were added to the plate and incubated for 1 h at RT. The plates were washed, and then, streptavidin-horseradish peroxidase (HRP) was added for 20–30 min at RT. The plates were washed again, the substrate was added, and the OD at 450 nm was measured. Kd (nM) was calculated using the following equation: ED50 (ng/mL)/molecular weight of binding protein.

### Adhesion assay

BSA (20 µg/ml) or TGFBI was coated onto 96-well plates and incubated for 2 h at 37 °C. Then, 3T3-L1 preadipocytes were pretreated with Notch-1 antibody (5 µg/ml) or control IgG for 15 min at 37 °C and plated at a density of 3,000 cells per well. After incubation for 30 min at 37 °C, the cells were washed and fixed with 4% paraformaldehyde in PBS. Adherent cells were imaged under a microscope and counted.

### Immunofluorescence staining

Recombinant TGFBI or galectin-3 was coated onto 6-well chamber slides, which were maintained for 24 h at 4 °C. Next, 3T3-L1 preadipocytes were plated into precoated wells and incubated. The following day, the cells were fixed in 4% PFA, permeabilized with 25% Triton X-100, and blocked with blocking buffer containing 1% BSA in PBS. The cells were incubated with a Notch-1-specific primary antibody (ab52627) for 2 h at RT and stained with a secondary antibody (Alexa Fluor^®^ 488) under the same conditions. The samples were then mounted using DAPI-containing mounting solution (Vector Laboratories, Burlingame, CA, USA) and imaged using a Leica DM IL LED microscope (Leica, Germany).

### Quantitative real-time polymerase chain reaction (qRT‒PCR)

RNA extraction, complementary DNA (cDNA) synthesis, and quantitative real-time PCR (qPCR) were performed as previously described^11^. PCR was performed on an iCycler iQ™ Real-Time PCR Detection System (Bio-Rad Laboratories, USA) using SYBR Green Master Mix (TOYOBO, Japan). The relative gene expression levels were determined using the 2^−ΔΔCt^ method and are expressed as the fold change increase compared with that of wild-type mice. The primer sequences are listed in the Supplementary Table; β-actin was used as the control gene.

### Western blot analysis

Protein extraction and western blot analyses were performed as previously described^21,22^. Briefly, total protein samples were separated by SDS‒PAGE and transferred to nitrocellulose membranes. The following primary antibodies were used: anti-GLUT-2 (Cat. sc-9117, Santa Cruz Biotechnology, Santa Cruz, CA, USA), anti-PGC-1α (Cat. ab54481; Abcam, Cambridge, UK), anti-PPARγ (Cat. ab19481; Abcam), anti-UCP-1 (Cat. UCP11-A; Alpha Diagnostics, San Antonio, TX, USA), anti-cleaved Notch-1 (Cat. D3B8; Cell Signaling Technology (CST), Danvers, MA, USA), anti-Notch-1 (Cat. ab52627; Abcam), anti-Hes-1 (Cat. D6P2U; CST), anti-C/EBPα (Cat. ab15048; Abcam), anti-tubulin (Cat. 2148; CST), anti-lamin B1 (cat. 12586; CST), and anti-β-actin (Cat. sc-47778; Santa Cruz Biotechnology) antibodies. The signals were quantified using a Fusion Solo Detector (Vilber Lourmat, Marne La Vallee, France).

### Immunoprecipitation

First, 3T3-L1 preadipocytes were treated with recombinant TGFBI (20 µg/ml) for 24 h, and total protein was extracted in RIPA buffer containing 10 mM N-ethylmaleimide (EMD Millipore, Darmstadt, Germany). Immunoprecipitation was carried out overnight at 4 °C with primary antibody, followed by the addition of Protein G agarose beads and incubation for 1 h. Beads were collected by pulse centrifugation and washed with lysis buffer. The precipitated proteins were analyzed by western blotting as described above.

### Immunohistochemistry

Inguinal white adipose tissue (iWAT) and liver tissue were fixed with 4% paraformaldehyde, processed, and embedded in paraffin. Tissue sections (5–7 μm thick) were stained with hematoxylin and eosin (H&E). The size of the adipocytes in three mice per genotype was measured using ImageJ software (NIH).

### Immunohistofluorescence (IHF)

iWAT was harvested from ND-fed or HFD-fed WT and KO mice. Tissues were fixed with 4% PFA and embedded in paraffin. The embedded tissues were cut into 4 μm-thick sections, heated at 60 °C, and multistained with DAPI, F4/80, and TGFBI. Multiplex immunofluorescence staining was performed as previously described^11^. Primary antibodies against TGFBI (ab189778, Abcam, dilution 1:100) and F4/80 (CST70076, CST, dilution 1:150) were stained and visualized using Opal 570 TSA and Opal 690 TSA, respectively. Nuclei were verified by DAPI staining. For quantification of single- or double-stained cells, 3–4 sections from three mice per experimental group were selected and counted.

### Statistical analysis

Statistical analyses for all experiments were performed using SPSS ver.20.0 (SPSS, Inc., Chicago, IL, USA). Statistical differences between groups were analyzed using a two-sided t test, and statistical significance was set at *p* < 0.05.

## Results

### TGFBI KO mice exhibit resistance to high-fat diet-induced obesity

**Fig. 1:**
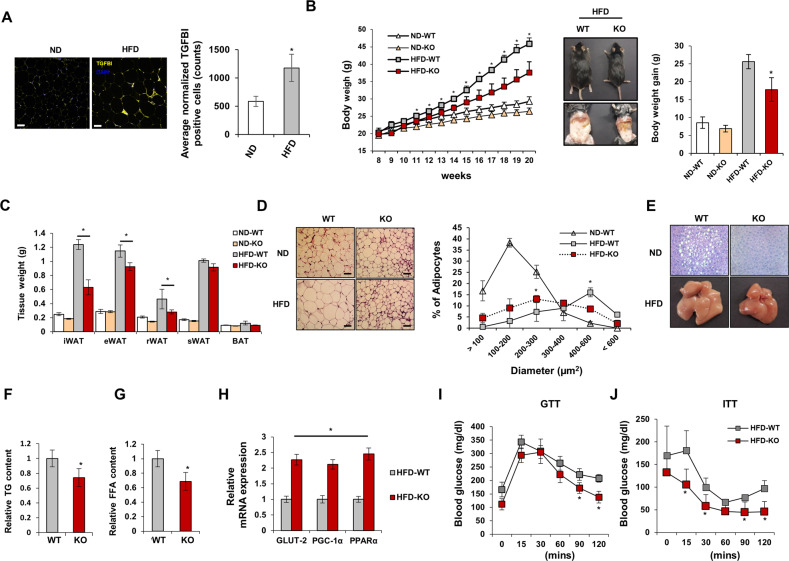
TGFBI-deficient mice are resistant to high-fat diet-induced obesity. **A** TGFBI was stained in iWAT from ND-fed or HFD-fed mice and quantified (right). Scale bar, 40 µm. **B** WT and KO mice (8 weeks old) were fed a high-fat diet (HFD) or normal diet (ND) for 12 weeks, and body weight was recorded every week (left). On the final day of the experiment, representative photos were taken of the HFD-fed WT and KO mice (middle). The gain in body weight was calculated using the following formula: final weight (20 weeks old) – initial weight (8 weeks old). **C** Indicated organ weights from the above mice. **D** Representative H&E staining of iWAT obtained from the ND- or HFD-fed WT and KO mice. Scale bar, 50 µm. (left). Diameter distribution of adipocytes in the indicated adipose tissues (right). **E** Representative H&E staining of livers from the 20-week-old HFD-fed WT and KO mice. The bottom panel shows representative livers from the above mice. **F-G** TG and FFA content from the HFD-fed WT and KO mice. **H** Relative mRNA expression of the indicated genes in the livers of the HFD-fed WT and KO mice. **I-J** GTT and ITT results. Error bars represent the ± SEM. *p < 0.05 by two-sided t test.

A genetically modified animal model was used to determine whether TGFBI affected diet-induced obesity. The PCR analysis confirmed the deletion of the TGFBI gene, and the 376-bp product was compared with the 1099-bp product for the WT mice (Supplementary Fig. 1b). The deletion of TGFBI in the plasma of the WT and KO mice was also confirmed by ELISAs. We assessed the overaccumulation of TGFBI in the adipose tissue of the HFD-fed obese mice and compared it with that in the ND-fed lean mice (Fig. 1A). The whole body weight of 11-week-old HFD-fed KO mice was significantly lower than that of WT mice of the same age (Fig. 1B). The 20-week-old HFD-fed KO mice showed a dramatic decrease in body weight gain compared with the WT mice. These differences were also observed between the ND-fed WT and KO mice, although a smaller difference was observed compared with that for the HFD-fed groups. A significant decrease was observed in the inguinal WAT (iWAT), retroperitoneal WAT (rWAT), epididymal WAT (eWAT), and liver mass of the HFD-fed KO mice, whereas the brown adipose tissue (BAT) and subcutaneous WAT (sWAT) mass were unaltered compared with those of the HFD-fed WT mice (Fig. 1C). The masses of the heart, lungs, kidneys, and spleen tissue were comparable between the WT and TGFBI KO mice (Supplementary Fig. 1C). As expected, with suppressed adipose expansion, the size of the iWAT adipocytes decreased in the HFD-fed KO mice compared with that of the HFD-fed WT mice (Fig. 1D). These results suggest that TGFBI KO mice exhibit resistance to HFD-induced body weight gain and adipose tissue expansion.

HFD-induced ECM remodeling was apparent not only in adipose tissue but also in the liver, and HFD-fed mice exhibited increased expression of collagen type I and III in the liver^18,23^. This remodeling process was also closely associated with insulin resistance^24^. Therefore, we determined whether the lack of TGFBI contributed to improved homeostasis and liver function in HFD-fed mice. The HFD-fed KO mice exhibited a marked reduction in lipid composition, triacylglycerol (TG) content, and free fatty acid (FFA) content in the liver compared with the HFD-fed WT mice (Fig. 1E–G). The HFD-fed KO mice showed increased expression of gluconeogenesis-related genes, including those encoding GLUT-2, PGC-1α, and PPARα (Fig. 1H). Glucose tolerance and insulin sensitivity improved in the HFD-fed KO mice compared with those in the HFD-fed WT mice (Fig. 1I–J). To determine whether differences occurred between sexes, we evaluated the phenotype of HFD-fed female KO mice. Similar to the HFD-fed male KO mice, the female KO mice also exhibited lower body weight and iWAT mass and better glucose homeostasis than the female WT mice (Supplementary Fig. 2a–f). TGFBI expression was highly upregulated in the iWAT, liver, and muscle and increased with HFD-induced progressive weight gain (Supplementary Fig. 1d, e). However, TGFBI expression was not significantly altered between the groups in other types of adipose tissue, such as eWAT, rWAT, and BAT. Therefore, we focused on the possible reasons for altered TGFBI levels in iWAT. Taken together, these results suggest that TGFBI KO mice had an improved capacity for obesity-induced metabolic changes.

### TGFBI KO induces the expression of proteins involved in the PPARγ signaling pathway

**Fig. 2:**
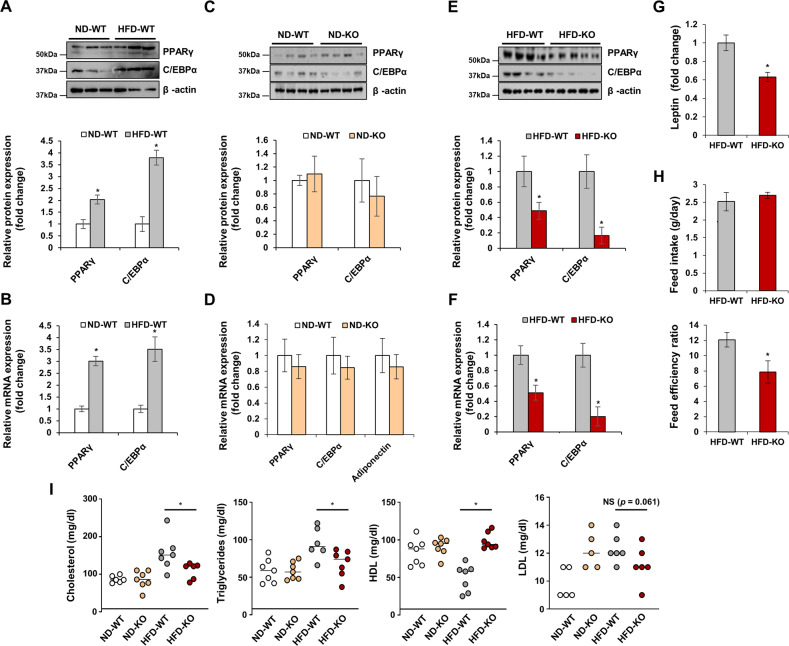
HFD-fed TGFBI-deficient mice show reduced expression of adipogenic genes. **A-F** Protein and mRNA expression of the indicated genes in iWAT obtained from ND-fed (**A-B**) or HFD-fed (**C-D**) WT and KO mice. The expression levels were normalized to those of β-actin. **G** Relative plasma leptin levels of the HFD-fed WT and KO mice. **H** Feed intake was calculated using the following formula: total feed intake (entire experimental period)/days (entire experimental period, 83 days). The feed efficiency ratio was calculated using the following formula: body weight gain (g)/amount of feed provided (g) × 100. **i** Plasma cholesterol, TG, HDL (high-density lipoprotein), and LDL (low-density lipoprotein) were determined in the ND-fed or HFD-fed WT and KO mice. Error bars represent the mean ± SEM. *p < 0.05 by two-sided t test.

To explore the mechanisms underlying the protective effects of TGFBI during obesity, we examined the PPARγ signaling pathway, which is closely involved in adipocyte differentiation, in iWAT from ND- and HFD-fed WT and TGFBI KO mice. The mRNA and protein expression of PPARγ and C/EBPα was increased in iWAT from 20-week-old HFD-fed WT mice compared with that in iWAT from the corresponding controls (Fig. 2A, B). We observed that the protein and mRNA expression levels of PPARγ and C/EBPα did not change in the ND-fed WT and KO mice (Fig. 2C, D). Interestingly, the protein and mRNA expression levels of PPARγ and C/EBPα were significantly lower in the HFD-fed TGFBI KO mice than in the WT mice (Fig. 2E, F). In addition, the serum leptin levels were decreased in the TGFBI KO mice (Fig. 2G). Notably, the TGFBI KO mice showed improved food efficiency, although their food intake was comparable with that of the WT mice (Fig. 2H). These results indicated that the obesity resistance of the TGFBI KO mice was not affected by the reduction in fat in the diet. In addition, plasma lipids, such as cholesterol and TG, were dramatically reduced in the HFD-fed TGFBI KO mice compared to the WT mice, while an opposite result was observed for HDL (Fig. 2I). Plasma LDL levels did not differ considerably between the WT and TGFBI KO mice. Taken together, these results demonstrate that TGFBI deficiency may lead to the suppression of excess adipose expansion and blood lipid accumulation, possibly via the PPARγ signaling pathway.

### TGFBI KO mice exhibit oversecretion of adipsin and higher macrophage populations

**Fig. 3:**
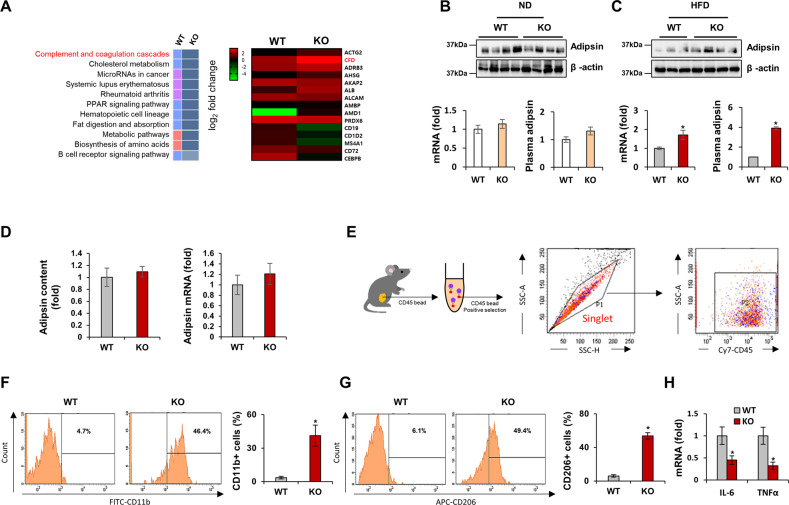
TGFBI KO mice show increased adipsin expression and macrophage infiltration in adipose tissue. **A** Gene set enrichment (left) and expression heatmap (right) in iWAT obtained from 20-week-old HFD-fed WT and KO mice. **B-C** Adipsin expression levels were verified by performing western blot analysis (upper), qRT‒PCR (bottom, left), and ELISAs (bottom, right) in iWAT obtained from the ND-fed (B) and HFD-fed (C) WT and KO mice. **D** Secretory (left) and mRNA expression of adipsin (right) in differentiated BM-MSCs isolated from the WT and KO mice. **E** Cy7-CD45-positive cells were isolated from iWAT of the WT and TGFBI KO mice and identified by FACS. **F-G** FITC-CD11b- **(F)** and APC-CD206-positive **(G)** cells were verified and quantified (right). **H** Relative mRNA levels of the indicated genes in iWAT obtained from the above mice. Error bars represent the ± SEM. *p < 0.05 by two-sided t test.

To further elucidate the mechanism through which metabolism benefits HFD-fed TGFBI KO mice, we performed an RNA sequence analysis of iWAT from these mice (Fig. 3A). Among the gene processes that were modulated in the TGFBI KO mice, the significantly enriched complement and coagulation cascades were examined. Adipsin (Complement Factor D, CFD) is a regulatory protein of the complement cascade whose expression is downregulated in mice with acquired metabolic diseases^25^. Consistently, the expression of adipsin was reduced in the HFD-fed obese mice compared with the ND-fed normal mice (Supplementary Fig. 3a, b). We observed that the mRNA, protein, and secretory levels of adipsin were increased in the HFD-fed TGFBI KO mice, whereas no difference was observed between the ND-fed WT and KO mice (Fig. 3B, C).

Next, we hypothesized that primary adipocyte-derived BM-MSCs could alter adipsin secretion in an autocrine manner. However, the adipsin levels of differentiated BM-MSCs were comparable between those isolated from the TGFBI KO mice and those isolated from the WT mice (Fig. 3D). Previous studies have indicated that fat-produced adipsin regulates inflammation and immune status in adipose tissues and rear ankle^26,27^. To determine the contribution of immune cells, such as macrophages, to the processes observed in the present study, we measured the macrophage population in adipose tissues. CD45 + hematopoietic cells in adipose tissues were sorted using immunomagnetic bead separation (Fig. 3E). The populations of CD45+/CD11b+ and CD45+/CD206+ M2 macrophages were decreased in iWAT from the HFD-fed mice compared with iWAT from the ND-fed mice (Supplementary Fig. 4a, b). These M2 macrophages were substantially enriched in iWAT from the TGFBI KO mice, similar to that in iWAT of the ND-fed mice (Fig. 3F–G). In addition, the levels of the proinflammatory cytokines interleukin (IL)-6 and tumor necrosis factor (TNF)-α were decreased in the TGFBI KO mice (Fig. 3H). These results suggest that TGFBI facilitates the polarization of macrophages toward an anti-inflammatory M2 phenotype in adipose tissue.

### Adipose macrophages regulate the browning and adipsin secretion of adipocytes

**Fig. 4:**
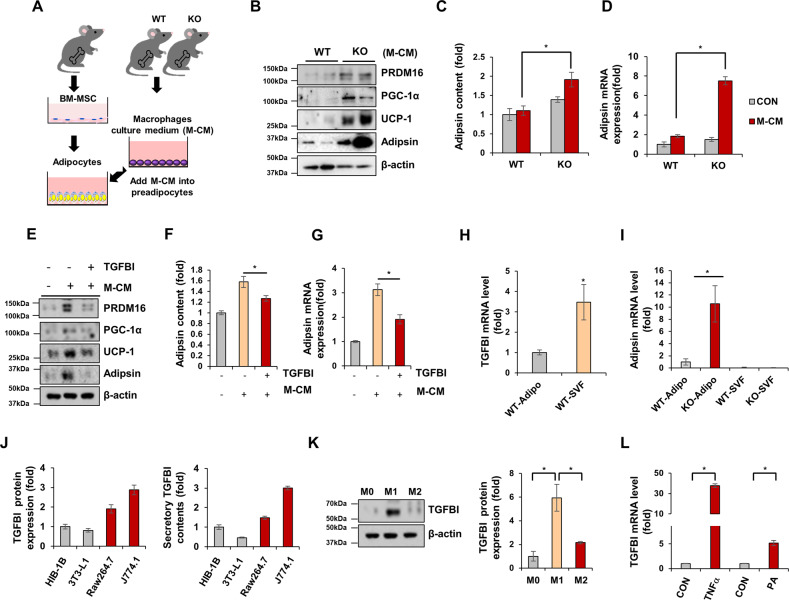
TGFBI-deficient macrophages induced increased adipsin and browning-related protein expression on adipocytes in a paracrine manner. **A** Schematic of the experimental design. BM-MSCs were isolated from WT mice and induced to differentiate with M-CM obtained from WT or KO mice. **B** Expression of the indicated proteins in differentiated BM-MSCs cultured with WT or KO macrophages. **C-D** Secretory and mRNA expression of adipsin in the above cells. **E-G** BM-MSCs were either unstimulated or treated with 10 µg recombinant TGFBI under coculture conditions with TGFBI KO macrophages. Expression of the indicated proteins (**E**) and secretory (**F**) and mRNA (**G**) levels of adipsin in the above cells. **H–I** Adipocytes and SVFs were isolated from iWAT obtained from WT and KO mice. The mRNA expression of TGFBI (**H**) and adipsin (**I**) was measured in the indicated fractions. **J** TGFBI expression in differentiated HIB-1B and 3T3-L1 adipocytes and Raw264.7 and J774.1 macrophages (M1 phenotype) was assessed by western blot analysis (left) and ELISAs (right). **K** Human THP-1 macrophages were treated with 10 ng/mL LPS and 20 ng/mL IFN-γ or 20 ng/mL IL-4 and 20 ng/mL IL-13 for polarization into M1 and M2 macrophages, respectively. TGFBI expression was verified and quantified (right panel). **L** THP-1 macrophages were treated with rhTNFα (20 ng/mL) and palmitic acid (200 µM). TGFBI mRNA expression was verified. Error bars represent the ± SEM. *p < 0.05 by two-sided t test.

Because increased numbers of macrophages were present in TGFBI KO mice, we sought to determine whether adipsin expression was regulated by macrophages under indirect coculture conditions (Fig. 4A). We found that adipocytes cultured with the TGFBI KO macrophages contained brown-like adipocytes that featured multiple smaller lipid droplets in their cytoplasm (Supplementary Fig. 5a). Consistent with these morphological differences, PRDM16, PGC-1α, UCP-1, and adipsin were mainly detected in adipocytes cocultured with macrophages isolated from TGFBI KO mice compared with those isolated from WT mice (Fig. 4B–D and Supplementary Fig. 5b). Therefore, in this study, we focused on adipose browning. We induced adipocyte differentiation into brown-like adipocytes using T3 and rosiglitazone (Rosi). Consequently, these brown-like adipocytes demonstrated higher UCP-1 and PGC-1α expression than normal MDI-treated adipocytes (Supplementary Fig. 6a, b). We confirmed the effects of TGFBI on adipsin and browning marker expression under macrophage coculture conditions. TGFBI treatment ameliorated adipsin, PRDM16, PGC-1α, and UCP-1 expression in adipocytes cultured with TGFBI KO macrophages (Fig. 4E–G). Interestingly, we observed that TGFBI was primarily secreted by the small vascular fraction containing macrophages rather than by adipocytes. In contrast, adipsin levels were significantly higher in adipocytes (Fig. 4h, i). TGFBI expression was further confirmed in HIB-1B and 3T3-L1 adipocytes and Raw 264.7 and J774.1 macrophage lines. Concordantly, TGFBI expression was higher in macrophage cell lines than in adipocyte cell lines (Fig. 4J). Furthermore, we analyzed TGFBI expression in macrophages polarized toward the M1 and M2 subtypes and observed that TGFBI expression was threefold higher in proinflammatory M1 macrophages than in anti-inflammatory M2 macrophages (Fig. 4K). TNFα and palmitic acid (PA) markedly increased TGFBI expression in macrophages (Fig. 4L). These results indicate that macrophages, particularly the M1 subtype, are the main source of TGFBI in adipose tissue and can be secreted in response to inflammatory stimuli, such as TNF-α and PA. Taken together, these results suggest that these macrophages play important roles in the regulation of TGFBI-mediated adipsin secretion from adipocytes. Additionally, TGFBI is largely derived from macrophages and may influence adipsin expression in adipocytes in a paracrine manner, which is closely associated with adipocyte characteristics and browning.

### TGFBI is mainly localized in ATMs, and its loss leads to the secretion of unique cytokines

**Fig. 5:**
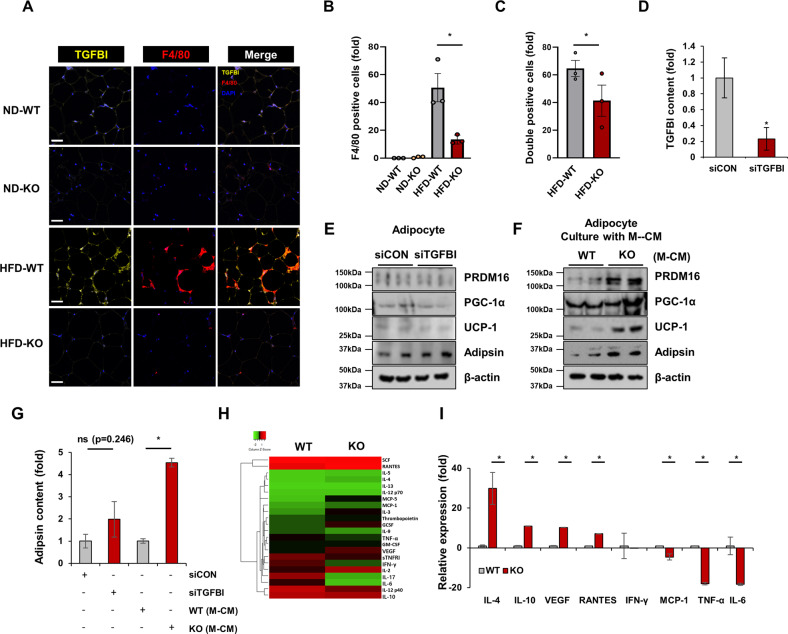
TGFBI KO BM-DMs secrete unique cytokines. **A-C** TGFBI, F4/80, and DAPI were multistained in iWAT from ND-fed or HFD-fed WT and KO mice. F4/80 single-positive **(B)** or F4/80 and TGFBI double-positive **(C)** cells were counted in whole tissue from three individual mice per experimental group. **D-F** 3T3-L1 preadipocytes were transfected with lentivirus encoding either TGFBI or control siRNA. After infection, the cells were induced to differentiate into mature adipocytes, and the expression of the indicated proteins was verified using western blot analysis **(D)**. Parental 3T3-L1 adipocytes were cocultured with either WT or TGFBI KO BM-DMs **(F)**. The indicated proteins were assessed using western blotting **(F)** and ELISAs **(G)**. **H-I** Expression heatmap showing changes in cytokines secreted by BM-DMs in the WT and TGFBI KO mice (*n* = 3). The expression level of each cytokine is shown in a green (low expression) and red (high expression) color scheme. Error bars represent ± SEM. *p < 0.05 by two-sided t test.

Given the main source of TGFBI, we assessed the localization of TGFBI in iWAT. As previously established, TGFBI was overexpressed with F4/80 (Fig. 5A, B). Importantly, we found colocalization of TGFBI and F4/80 in the HFD-fed WT mice compared to the ND-fed mice (Fig. 5C). Next, we determined whether TGFBI played a role in adipocyte browning in 3T3-L1 adipocytes. We also observed no difference in the expression of PRDM16, PGC-1α, UCP-1, and adipsin between the TGFBI knockdown (siTGFBI) and control (siCON) cells (Fig. 5D, E). In contrast, adipocyte browning-inducing effects were observed in adipocytes cocultured with macrophages (Fig. 5F). The expression of PRDM16, PGC-1α, UCP-1, and adipsin was increased in the parental adipocytes cultured with TGFBI KO macrophages compared to that in the normal adipocytes cultured with WT macrophages. Elevated adipsin levels were further confirmed using ELISAs (Fig. 5G). These results were consistent with our observation of adipose browning in the BM-MSCs cocultured with TGFBI KO BM-DMs (Fig. 4).

Next, to identify factors secreted by TGFBI KO macrophages that may affect adipose browning, we performed a cytokine array to assess cytokines released by WT and TGFBI KO BM-DMs. Among all the tested factors, IL-4, IL-10, VEGF, regulated upon activation normal T cell expressed and secreted (RANTES), monocyte chemoattractant protein-1 (MCP-1), TNF-α, and IL-6 were differentially expressed in cell culture supernatants of the TGFBI KO BM-DMs and the WT BM-DMs (Fig. 5H, I). IL-4, IL-10, VEGF, and RANTES were upregulated, whereas MCP-1, TNF-α, and IL-6 were downregulated in the cell culture supernatants of the TGFBI KO BM-DMs compared to those in the cell culture supernatants of the WT BM-DMs. These findings suggest that these cytokines might contribute to adipose browning and the tissue microenvironment in TGFBI KO mice.

### TGFBI induces Notch-1 signaling activation in adipocytes

**Fig. 6:**
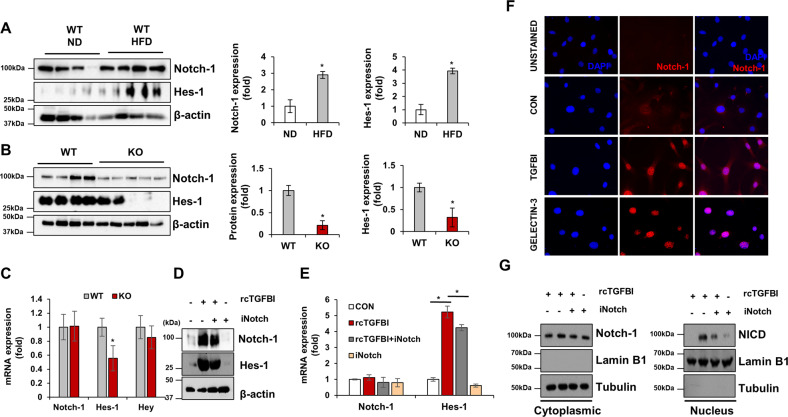
TGFBI regulates the activation of Notch-1 in adipocytes. **A** Notch-1 and Hes-1 protein expression in iWAT obtained from ND- and HFD-fed WT mice. **B, C** Protein and mRNA expression of the indicated genes in iWAT obtained from the HFD-fed WT and KO mice. **D-E** The 3T3-L1 adipocyte line was treated with SHAM1 (Notch-1 inhibitor) in the presence or absence of recombinant TGFBI. **F** Localization of Notch-1 in 3T3-L1 cells treated with either recombinant TGFBI or galectin-3. **G** Indicated protein expression in the cytoplasm (left) and nucleus (right).

The Notch-1-induced transcriptional regulator Hes-1 downregulates adipsin expression in adipocytes^28^_,_ and Notch-1 signaling promotes obesity progression and ameliorates adipose browning^29^. However, the mechanisms underlying the activation of these processes and whether these processes are related to ECM remodeling in HFD-induced obese mice have not been clarified. HFD feeding induced the overexpression of Notch-1 and Hes-1 in WAT obtained from the WT mice compared with that from the lean mice (Fig. 6A, B). Interestingly, Notch-1 protein expression was significantly reduced in the TGFBI KO mice, whereas differences at the mRNA level relative to that in the WT mice were not observed (Fig. 6C). In contrast, Hes-1 mRNA and protein levels decreased by 1.5- to 2.0-fold in the TGFBI KO mice. To determine whether exogenous treatment with TGFBI affected Notch-1 activation, we measured the expression of Notch-1 and Hes-1 in TGFBI-treated adipocytes. TGFBI treatment increased the protein expression of Hes-1 and Notch-1 in 3T3-L1 adipocytes (Fig. 6D). Consistent with the in vivo data, downregulation of Hes-1 mRNA expression was observed, while changes in Notch-1 expression were not observed (Fig. 6E). These data suggest that TGFBI controls Notch-1 abundance at the post-transcriptional level.

Next, we postulated that TGFBI may facilitate the nuclear translocation of Notch-1. The results showed that TGFBI treatment induced nuclear translocation of Notch-1, which was similar to that observed for the positive control galectin-3 (Fig. 6F). Furthermore, the content of the Notch-1 intracellular domain increased in the nuclear fractions of TGFBI-treated 3T3-L1 adipocytes, whereas Notch-1 activation was blocked by a Notch-1 inhibitor in cells cotreated with TGFBI (Fig. 6G). Furthermore, Notch-1 expression remained unchanged in the cytoplasmic fractions. These results suggest that TGFBI activates the nuclear translocation of Notch-1 in adipocytes.

### Notch-1 binds to TGFBI and regulates cell adhesion of adipocytes

**Fig. 7:**
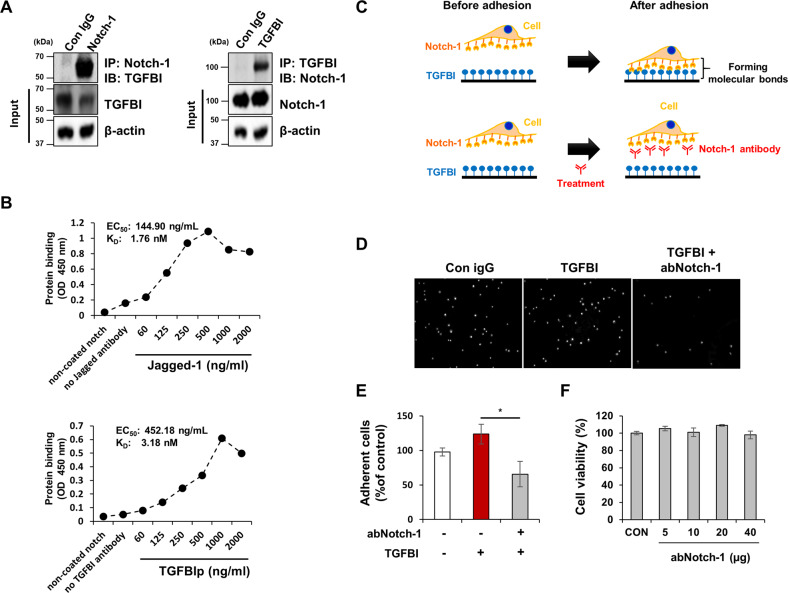
TGFBI physiologically interacts with Notch-1. **A** IP analysis of the interaction of Notch-1 and TGFBI. **B** Non-cell-based binding assay. Recombinant Notch-1 protein was precoated and allowed to bind to TGFBI or Jagged-1 at the indicated concentrations. **C** Schematic of the experimental design for the cell adhesion assay. **D-E** Pretreatment of 3T3-L1 adipocytes with either control IgG or Notch-1-specific antibody (abNotch-1) was performed, and the cells were allowed to adhere to recombinant TGFBI-coated plates. **F** Cytotoxicity of abNotch-1 at the indicated concentrations was tested using an MTT assay. Error bars represent the ± SEM. *p < 0.05 by two-sided t test.

Next, we determined whether TGFBI binds to Notch-1 in 3T3-L1 adipocytes. Notch-1 was physically associated with TGFBI in 3T3-L1 adipocytes (Fig. 7A). In a non-cell-based system, TGFBI exhibited binding affinity for Notch-1 (Kd = 3.18 nM), although its affinity was weaker than that of Jagged-1, which is a strong ligand for Notch-1 (Fig. 7B). The ECM facilitates cellular attachment and mediates cellular mobility, adhesion, proliferation, and differentiation^30^. To determine whether TGFBI controls adipocyte adhesion via Notch-1, we performed a cell adhesion assay using TGFBI-coated plates (Fig. 7C). Notch-1 inhibition markedly suppressed the adhesion capacity of 3T3-L1 adipocytes on TGFBI-coated plates, although cytotoxicity was not observed (Fig. 7D–F). These results indicated that TGFBI is functionally active and directly binds to Notch-1 in adipocytes.

### TGFBI suppresses adipocyte browning and adipsin secretion by activating Notch-1 signaling

**Fig. 8:**
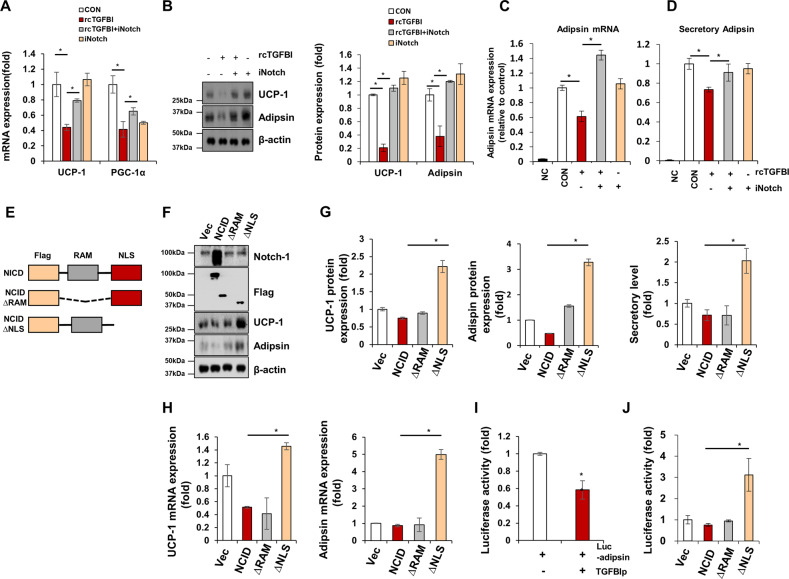
Notch-1 represses adipsin promoter activity and browning-related protein expression. The 3T3-L1 adipocyte line was treated with Notch-1 inhibitor throughout the differentiation period in the presence or absence of recombinant TGFBI. **A,B** mRNA and protein expression of the indicated genes. **C, D** Adipsin mRNA and secretory levels of each indicated group. **E** The 3T3-L1 adipocytes were transfected with the indicated WT and mutant Notch-1 (RAM and NLS). **F-H** Protein **(F-G)** and mRNA **(H)** expression of the indicated genes in transfected cells. **I** HEK293 cells were transfected with an Adipsin-Luc vector (−430 bp) in the presence or absence of recombinant TGFBI. **J** HEK293 cells were cotransfected with the indicated Notch-1 construct and Adipsin-Luc vector. Error bars represent the ± SEM. *p < 0.05 by two-sided t test.

To establish the physiological importance of TGFBI, we examined whether it regulates adipocyte browning by activating Notch-1. Notably, this TGFBI-induced reduction was restored by cotreatment with a Notch-1 inhibitor and significantly elevated the expression of UCP-1 and PGC-1α (Fig. 8A, B). Accordingly, adipsin expression and secretion were significantly elevated by cotreatment of the cells with the Notch inhibitor and TGFBI compared to treatment with TGFBI alone (Fig. 8C, D). Moreover, the adipsin levels were unchanged by treatment with the Notch-1 inhibitor alone. These findings indicate that TGFBI ameliorates adipsin-mediated browning via a Notch-1-dependent mechanism. Finally, we investigated whether Notch-1 regulated the expression of adipsin and browning markers. Notch-1 has intracellular domains that include the RAM (Rbpj interacting domain) and NLS (nuclear localization signal) (Fig. 8E). Deletion of the NLS domain upregulated the mRNA and protein expression of UCP-1 and adipsin (Fig. 8F–H), while deletion of RAM did not induce the expression of these proteins. The promoter activity of adipsin was repressed by TGFBI (Fig. 8I); however, this effect was reversed by deleting the NLS domain in the presence of TGFBI (Fig. 8J). These results indicate that deletion of the NLS domain directly enhances adipsin promoter activity and markedly increases the expression of browning-related genes.

## Discussion

Obesity is characterized by abnormal adipose expansion and fibrosis accompanied by the overaccumulation of ECM^31^. A recent study indicated that remodeling is required to maintain a healthy adipose tissue condition^32^. However, limitations have been observed in studies that primarily focused on major ECM proteins, such as collagen and fibronectin. TGFBI has been implicated in the pathogenesis of cancer and diabetic retinopathy^33,34^. Moreover, previous studies have detected TGFBI in adipose tissue and associated TGFBI polymorphisms with insulin levels and body mass^11,35^. However, the gain- and loss-of-function mechanisms of TGFBI are not completely understood. In the present study, we demonstrated that the ECM component TGFBI plays a role in HFD-induced obesity and improves several physiological conditions, such as glucose/insulin resistance, adipose expansion, liver steatosis, and adipocyte differentiation.

Indeed, weight loss resulting from TGFBI deficiency occurs not only in HFD-fed mice but also in ND-fed mice. A previous study demonstrated that TGFBI regulates periosteal bone formation, including bone mass, size, and strength^36^. TGFBI KO mice show reduced bone mass and 8–20% lower body weight than WT mice during postnatal development. However, why TGFBI KO mice exhibit a slight decline in body weight gain due to ND feeding remains unclear. In the present study, we found that HFD-fed TGFBI KO mice exhibited a greater reduction in adipose and liver mass and lipid accumulation. Future studies are warranted to address the tissue-specific and direct targeted effects of TGFBI using engineered mice.

In the present study, a higher relative expression of TGFBI in response to HFD was observed in iWAT but not in other adipose tissues of overweight mice. iWAT has been widely studied with respect to adipose browning because it exhibits higher browning than other types of adipose tissues^37^. Therefore, we suggest that TGFBI contributes to specific adipose tissue and adipocyte characteristics, ultimately affecting whole-body metabolism and obesity.

TGFBI overexpression is detected in fibroblasts, peritoneal cells, macrophages, and T cells^38,39^, among which macrophages are predominant producers of TGFBI. In the present study, we found that TGFBI was largely produced by small vascular fractions containing various immune cells and secreted from adipocytes. TGFBI expression was higher in macrophage cell lines than in adipocyte cell lines. TGFBI knockdown reportedly accelerated human preadipocyte adipogenesis^40^, suggesting that TGFBI acts on adipocytes in an autocrine manner. However, no explanation was provided for the effect of TGFBI on adipose browning or the underlying mechanisms. In the present study, we did not observe significant differences in adipsin expression between adipocytes cultured alone or those isolated from WT mice following TGFBI KO. These results suggest that additional metabolic stimulatory conditions or factors, such as IL-4, IL-10, and VEGF, assessed in the cytokine array for BM-DMs might contribute to adipsin secretion from adipocytes. M2-polarized macrophages secrete VEGF and IL-10, which play a crucial role in angiogenesis, an essential process for WAT browning^41,42^. Several cytokines reportedly play essential roles in adipose browning and metabolic function^43,44^. VEGF deletion reduced vasculature, increases hypoxia and inflammation in white adipose tissue and subsequently induces metabolic deterioration^45^. In contrast, VEGF overexpression induced adipose angiogenesis and other opposing effects in adipose tissues. Consistent with these findings, our results provide several lines of evidence supporting the notion that polarized macrophages obtained from TGFBI KO mice regulate the expression of adipose browning-related markers and adipsin in adipocytes, possibly by controlling the secretion of cytokines, such as IL-4, IL-10, and VEGF. Although we did not determine whether these cytokines could directly regulate adipocyte browning and adipsin expression in the present study, we propose that TGFBI deletion induces adipose browning by mainly acting on adipose macrophages, possibly because of the changes in macrophage cytokine secretion.

Macrophages are the primary inflammatory cells found in inflamed adipose tissue that control adipose homeostasis and energy expenditure^46,47^. Several reports have indicated the involvement of cytokines secreted from adipose tissue macrophages in beige adipocyte formation and WAT browning^48^. Consistent with these findings, our results provided several lines of evidence indicating that adipose-associated macrophages regulate the expression of inflammatory cytokines and browning markers in adipose tissues obtained from TGFBI KO mice. We speculate that the different phenotypes detected in our study occurred in a distinct population of CD11b + and CD206 + M2-like macrophages in TGFBI KO mice compared to those in WT mice.

Interestingly, adipsin levels were strongly elevated in adipose tissue and adipocytes cocultured with macrophages isolated from TGFBI KO mice. We confirmed that adipsin expression was positively correlated with the expression of the adipose browning markers UCP-1 and PGC-1α. In TGFBI KO mice, adipsin was secreted in response to macrophages; thus, we concluded that increased adipsin expression is closely associated with adipose browning and other metabolic benefits in TGFBI KO mice.

The Notch-1 signaling pathway has been implicated in various biological processes, such as tumorigenesis, apoptosis, and proliferation^49^. Recently, Notch signaling was also found to be important in adipogenesis^50^. Inhibition of Notch-1 and its signaling mediators resulted in adipose browning and regulates adipsin expression^28,29^, which is consistent with our observation that the pharmacological effects and genetic mutations of Notch-1 resulted in elevated adipose browning. Furthermore, we discovered that Notch-1 represses adipsin expression and that the Notch-1 intracellular domain NLS is required for the inhibition of adipsin transcription. Notably, we discovered that TGFBI acts as a new ligand for Notch-1, and this signaling activation is required for suppression of adipsin secretion and adipose browning.

In summary, the present study revealed that the deletion of the ECM protein TGFBI has a protective effect against HFD-induced metabolic disorders. Mechanistically, TGFBI activates Notch-1 signaling and subsequently regulates adipsin expression, which may explain the upregulation of adipose browning-related gene expression and the beneficial metabolic effects observed in TGFBI KO mice. Taken together, our findings offer new insights for the development of new therapeutic approaches for obesity and related disorders via TGFBI regulation.

## Supplementary information

Supplemental infomration

Data Set 1
