# Supplementary material for: TGFBI remodels adipose metabolism by regulating the Notch-1 signaling pathway
Source: Exp Mol Med. 2023 Mar 1;55(3):520–31. doi: 10.1038/s12276-023-00947-9 (PMC10073093; doi:10.1038/s12276-023-00947-9)
Supplement: Supplementary file 1 — Supplemental infomration [file 12276_2023_947_MOESM1_ESM.docx]

**SUPPLEMENTARY INFORMATION**

**TGFBI remodels adipose metabolism by regulating the Notch-1 signaling pathway**

Seul Gi Lee^1,8^, Jongbeom Chae^1^, Seon Min Woo^8^, Seung Un Seo^8^, Ha-Jeong Kim^2^, Sang-Yeob Kim^3^, David D. Schlaepfer^4^, In-San Kim^5,6^, Hee-Sae Park^7^, Taeg Kyu Kwon^8,9*^, and Ju-Ock Nam^1,10*^

*^1^Department of Food Science and Biotechnology, Kyungpook National University, Daegu 41566, Republic of Korea*

*^2^Department of Physiology, School of Medicine, Kyungpook National University, Daegu 41944, Republic of Korea*

*^3^ASAN Institute for Life Sciences, ASAN Medical Center, Seoul 05505, Republic of Korea*

*^4^Moores Cancer Center, University of California, San Diego, La Jolla, CA 92093, USA*

*^5^KU-KIST Graduate School of Converging Science and Technology, Korea University, Seoul 02841, Republic of Korea*

*^6^Center for Theragnosis, Biomedical Research Institute, Korea Institute Science and Technology (KIST), Seoul 02792, Republic of Korea*

*^7^School of Biological Sciences and Technology, Chonnam National University, Gwangju 61186, Republic of Korea*

*^8^Department of Immunology, School of Medicine, Keimyung University, Daegu 42601, Republic of Korea*

*^9^Center for Forensic Pharmaceutical Science, Keimyung University, Daegu 42601, Republic of Korea*

*^10^Research Institute of Tailored Food Technology, Kyungpook National University, Daegu 41566, Republic of Korea*

*******Corresponding authors**

**Taeg Kyu Kwon**

Address*:* Keimyung University, 1095 Dalgubeoldaero, Dalseo-Gu, Daegu 42601, Korea.

Tel: +82-53-420-4811, Fax: +82-53-424-3349, E-mail: kwontk@dsmc.or.kr

**Ju-Ock Nam**

Address: Kyungpook National University, 80 Daehak-ro, Buk-gu, Daegu, Korea.

Tel: +82-53-950-7760, Fax: +82-53-950-7762, E-mail: namjo@knu.ac.kr

**Supplementary Fig. 1. Generation of TGFBI KO mice. a** Schematic illustration of the TGFBI knockout strategy. Exon 3 in TGFBI with a neomycin cassette was removed through Flp-mediated recombination. **b** Plasma TGFBI secretion from WT and TGFBI KO mice was detected using ELISA. **c** Indicated organ weights from the above mice. **d-e** The expression levels of TGFBI in different tissues of C57BL/6 mice. Error bars represent the ± SEM. *p < 0.05 by two-sided t-test.

**Supplementary Fig. 2. HFD-fed female TGFBI KO mice exhibit resistance to diet as male KO mice. a-b** Body weight and calculated weight gain in HFD-fed female WT and KO mice from 10 to 17 weeks of age (n = 10/group). **c-d** Adipose tissues (**c**) and organs (**d**) in 20-week-old HFD-fed female WT and KO mice. The right panels show representative photographs of the fat pads, liver, and abdomen (n = 10/group). **e-f** GTT and ITT results from 17-week-old HFD-fed female WT and KO mice. Error bars represent the ± SEM. *p < 0.05 by two-sided t-test.

**Supplementary Fig. 3. Adipsin expression is decreased by HFD feeding relative to shown in Fig. 4. a-b** Protein and mRNA expression of adipsin in iWAT obtained from ND- and HFD-fed WT mice. Error bars represent the ± SEM. *p < 0.05 by two-sided t-test.

**Supplementary Fig. 4. CD206+ and CD11b+ macrophages are depleted in HFD-fed mice. a-b** The CD45 positive cells were isolated the iWAT of 15-week-old ND- and HFD-fed mice. The CD45 labeled cells were stained with FITC-CD11b and APC-CD206 and verified by FACS. Error bars represent the ± SEM. *p < 0.05 by two-sided t-test.

**Supplementary Fig. 5. BM-MSCs cultured with TGFBI deficient macrophages exhibit a multilocular lipid phenotype and increased expression of browning-related genes. a** Representative images of BM-MSCs isolated from either WT or KO mice and cultured in presence or absence of M-CM obtained from either WT or KO mice. **b** mRNA expression levels of the indicated genes in these cells. Error bars represent the ± SEM. *p < 0.05 by two-sided t-test.

**Supplementary Fig. 6. Brown-adipocyte like feature of C3H10T1/2 induced by T3 and Rosiglitazone (Rosi).** **a-b** C3H10T1/2 cells were induced differentiation by MDI in the presence or absence of T3 and Rosi. (**a**) Representative images of differentiated C3H10T/12. (**b**) mRNA expression levels of the indicated genes in these cells. Error bars represent the ± SEM. *p < 0.05 by two-sided t-test.

| **Gene** | **Forward** | **Reverse** |
| --- | --- | --- |
| **Adiponectin** | GATGGCACTCCTGGAGAGAA | TCTCCAGGCTCTCCTTTCCT |
| **Adipsin** | CTGGGAGCGGCTGTATGT | CACGGAAGCCATGTAGGG |
| **C/EBPα** | AAGAAGACGGTGGACAAGCTG | TGCTCCACCTTCTTCTGCAGC |
| **GLUT-2** | ACTTGGAAGGATCAAAGCAATGT | CAGTCCTGAAATTAGCCCACAA |
| **Hes-1** | ACACCGGACAAACCAAAGAC | AATGCCGGGAGCTATCTTTC |
| **Hey** | CACCTGAAAATGCTGCACAC | ATGCTCAGATAACGGGCAAC |
| **IL-6** | TTCTTGGGACTGATGCTG | CTGGCTTTGTCTTTCTTGTT |
| **Leptin** | GGGCTTCACCCCATTCTGA | TGGCTATCTGCAGCACATTTTG |
| **Notch-1** | TAATGAGTGCAGCCAGAACC | CATAGGGCAGTTCACAGTGG |
| **PGC-1α** | CCCTGCCATTGTTAAGACC | TGCTGCTGTTCCTGTTTTC |
| **PPARα** | GCCTGTCTGTCGGGATGT | GGCTTCGTGGATTCTCTTG |
| **PPARγ** | GGAAGACCACTCGCATTCCTT | GTAATCAGCAACCATTGGGTCA |
| **TNFα** | TCCCTTTCACTCACTGGC | ACTTGGTGGTTTGCTACG |
| **UCP-1** | CTGCCAGGACAGTACCCAAG | TCAGCTGTTCAAAGCACACA |

**Supplementary Table. Primer sequence used in qRT-PCR.**
